# A case of splenic metastasis of ovarian cancer treated with complete laparoscopic splenectomy and transvaginal specimen extraction

**DOI:** 10.1186/s40792-016-0150-9

**Published:** 2016-03-14

**Authors:** Yoshiaki Takase, Naoki Tomizawa, Yasuaki Enokida, Takuya Shiraishi, Ryuji Katoh, Yujin Suto, Hiroaki Sato, Ken Muroya, Ryo Kurosaki, Katsumi Kobayashi, Kazuhisa Arakawa, Tatsumasa Ando, Izumi Takesyohi

**Affiliations:** Maebashi Red Cross Hospital, 3-21-36 Asahi-chou, Maebashi, Gunma 371-8511 Japan; Department of Thoracic and Visceral Organ Surgery, Gunma University Graduate School of Medicine, Maebashi, Gunma 371-0014 Japan

**Keywords:** Natural orifice specimen extraction, Transvaginal specimen extraction, Laparoscopic surgery, Splenectomy

## Abstract

A 61-year-old woman was diagnosed with right inguinal lymph node and splenic metastasis of ovarian serous cystadenocarcinoma. We performed right inguinal lymph node dissection and total laparoscopic splenectomy in the supine position followed by transvaginal specimen extraction (TVSE). First, using three ports, we extracted the right inguinal lymph node. We repaired the posterior wall of the inguinal canal using a mesh plug. We added two ports and displaced the spleen from the retroperitoneum and lifted it using a snake retractor, disconnecting the hilum using an automatic suturing device. Next, the posterior wall of the vagina was intraperitoneally incised. And an Alexis® laparoscopic system was inserted into the vagina. The cap maintained aeroperitoneum, a collection bag was inserted in the abdominal cavity via the vagina, and the spleen was collected. When the spleen was removed from the body, partial fragmentation of the organ was required in the bag. Organ fragmentation was performed only within the bag, and we made sure not to tear the bag. The vaginal wound was laparoscopically sutured. The patient had no operative complications and was able to actively ambulate at the first day after surgery due to a slight postoperative pain. Total laparoscopic splenectomy with TVSE in the supine position may be a safe and feasible method for selected female patients. This technique enables minimally invasive surgery for female patients with splenic disease.

## Background

Complete laparoscopic splenectomy is defined as a surgical technique in which all surgical maneuvers are performed through ports [1]. However, spleen extraction requires a small incision in the abdomen. To explore an even less invasive form of laparoscopic surgery, “natural orifice transluminal endoscopic surgery” (NOTES), in which the surgical maneuvers and organ extraction are performed through natural orifices without creating abdominal wounds, has been attempted [2, 3]. NOTES has the major advantage of circumventing the requirement for developing a small abdominal incision; however, NOTES still has technical issues and difficulties with regard to its clinical application [4]. “Natural orifice specimen extraction” (NOSE) techniques that are similar to NOTES have been attempted in recent years. Transvaginal specimen extraction (TVSE), one of these techniques, has been attempted mainly in Europe and the USA, and it is claimed to have the advantages of reducing surgical wound pain, reducing wound complications, shortening the period of hospitalization, and ensuring excellent esthetic outcomes [5–10].

Here, we describe a case in which we performed splenectomy in the supine position and extracted the specimen transvaginally to avoid abdominal incision. Widespread experience with laparoscopic pancreatectomies has allowed safe splenectomies in the supine position, by a safe and even less invasive surgical method using TVSE without the changing of body positions as required in conventional laparoscopic splenectomy [5–10]. The present surgical procedure was performed after obtaining approval from the Japanese Meabashi Red Cross hospital ethical committee.

## Case presentation

**Fig. 1:**
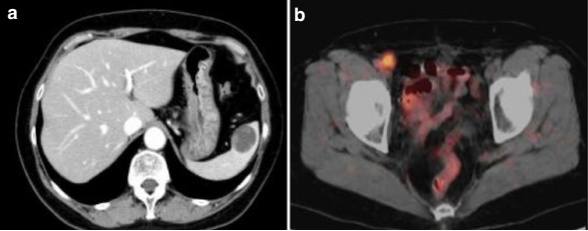
**a** Cystic lesion in the spleen (28 mm in diameter). **b** Enlarged right inguinal lymph node showing abnormal accumulation (MaxSUV = 5.0)

A 61-year-old woman underwent radical hysterectomy, pelvic lymphadenectomy, and omentectomy for ovarian serous cystadenocarcinoma at our hospital in July 2011. The pathological stage was pT3a, pN0, pM0 pStageIIIA according to the seventh classification of the International Union Against Cancer tumor-node-metastasis (TNM). She received six courses of adjuvant chemotherapy. However, in October 2013, she was diagnosed with right lymph node and splenic metastasis. A cystic lesion of 28 mm in diameter in the spleen and an enlarged lymph node of 16 mm in diameter in the right external iliac region were observed using abdominal computed tomography (CT; Fig. 1a). The enlarged right external iliac lymph node showed abnormal accumulation (MaxSUV = 5.0) in FDG-PET (Fig. 1b). There was no abnormal accumulation in the cystic lesion of the spleen or in any other organ. Her height was 149 cm, weight 53.7 kg, and BMI 24.2 kg/m^2^. The wound from the previous operation was observed in the median lower abdomen. Blood biochemical findings were not remarkable. Carbohydrate antigen 125 was within the normal range.

**Fig. 2:**
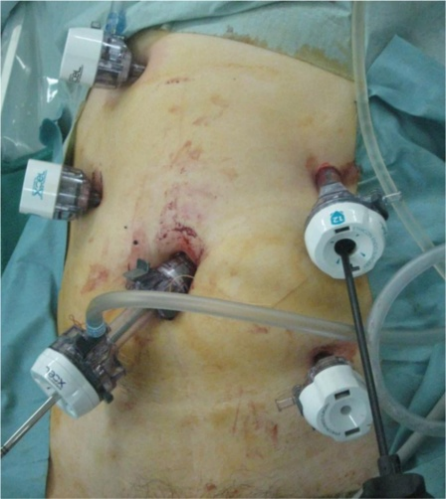
Port site for splenectomy

**Fig. 3:**
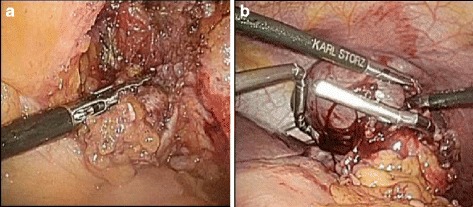
**a** Extraction of right inguinal lymph nodes. **b** Lifting the spleen using a snake retractor

**Fig. 4:**
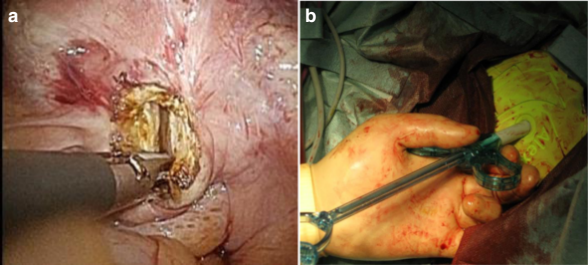
**a** Posterior colpotomy. **b** Alexis® laparoscopic system and Endo Catch^TM^ were inserted into the vagina

**Fig. 5:**
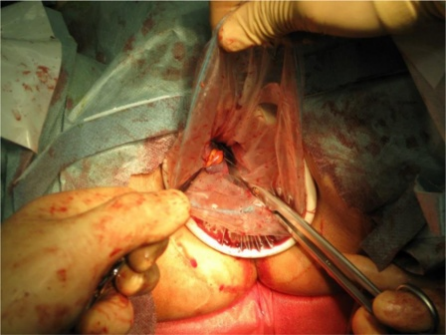
Transvaginal extraction

**Fig. 6:**
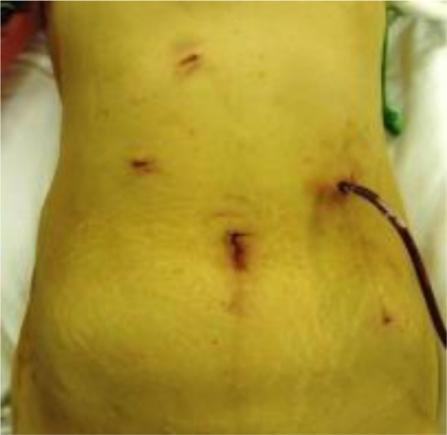
Postoperative scar

Before the operation, the vagina was washed with 500 mL of 0.02 % benzalkonium chloride solution in the lithotomy position. A 12-mm port was transumbilically inserted via laparotomy. A 5-mm port was inserted slightly lower to the left of the navel. Considering the need for subsequent laparoscopic splenectomy, a 5-mm port was inserted slightly upward to the right of the navel. There was almost no adhesion inside the pelvis, and the cervical stump was noted. There was a hard lymph node in the right groin near the inferior epigastric artery and vein and a whitish tumor in the lower pole of the spleen. The lymph node was extracted by making an incision in the peritoneum near the right groin. The inferior epigastric artery and vein were separated during the excision. Because the posterior wall of the inguinal canal became fragile, a ProLoop™ Mesh (Atrium, NH, USA) was inserted in the defect in the posterior wall of the inguinal canal, and the peritoneum was restored in accordance with the transabdominal preperitoneal approach (TAPP) (Fig. 3a). The excised lymph node was collected into a collection bag and extracted from the port in the umbilical region. With the patient still in the supine position, a 5-mm port in the upper right abdomen and a 12-mm port slightly to the upper left of the navel were added, making five ports in total (Fig. 2). The left gastroepiploic artery/vein and short gastric artery/vein were separated using LigaSure™ Vessel Sealing System (Valleylab, Boulder, Co, USA) to relieve the bursa omentalis. The spleen was mobilized from the retroperitoneum with the assistance of a snake retractor (Fig. 3b). The splenic hilum was separated using Endo GIA™ 45 mm, gray (Covidien, MA, USA) twice, and the splenectomy was completed. Next, the transvaginal route was established. We opened the pelvis with the patient in the Trendelenburg position, lifted the anterior vaginal wall ventrally using a gynecological Cusco, and confirmed the posterior vaginal fornix under direct vision and with a speculum. The posterior wall of the vagina was intraperitoneally incised using a SonoSurg (Olympus, Tokyo, Japan) (Fig. 4a), and an Alexis® laparoscopic system (Applied Medical, Rancho Santa Margarita, CA) was inserted into the vagina. The cap maintained aeroperitoneum, a collection bag (Endo Catch™, Covidien, MA, USA) was inserted in the abdominal cavity via the vagina, and the spleen was collected (Fig. 4b). When the spleen was removed from the body, partial fragmentation of the organ was required in the bag. Before fragmentation, we opened the bag via the vagina (Fig. 5). We had a direct view of the spleen, and we fragmented it to a minimum in the collecting bag using Kelly forceps and scissors. Organ fragmentation was performed only within the bag, and we made sure not to tear the bag. We changed our operation gowns and instruments after performing the procedure. It was possible to suture the vaginal incisional wound using laparoscopic 3-0 V-Loc™ (Covidien, MA, USA) after removing the Alexis® laparoscopic system, by maintaining aeroperitoneum via manual compression of the vaginal opening from the outside of the body by gauze and finger. After closing the wound, the Douglas pouch was thoroughly washed to confirm hemostasis. A drain tube was inserted under the left diaphragm through the 12-mm port in the upper left abdomen, and the port wound was dermo-stitched using an absorbable suture (Fig. 6). The operation time was 4 h and 27 min, and the blood loss was 300 g. The patient made satisfactory progress, had the drain removed on the fifth postoperative day, and was discharged on the sixth day. There was little pain, and she progressed positively into ambulation. Postoperative care of the incisional wound in the vagina was unnecessary. There was a cystic lesion in a portion of the extracted spleen. The cyst wall produced an enlarged tumor with profuse growth of cells with nuclear atypia. The extracted inguinal lymph node exhibited a histology similar to the previous ovarian cancer, and it was diagnosed as a metastasis of the ovarian cancer.

### Discussion

Here, we describe laparoscopic transvaginal splenectomy in a patient with splenic metastasis of ovarian cancer. To the best of our knowledge, this is the first report in the world about splenectomy using an extremely minimally invasive surgical method. A report on complete laparoscopic splenectomy has been published in 2013, but in that case, a hysterectomy was needed because of hypermenorrhea; therefore, the report focused on the use of the transvaginal route developed at the time of the hysterectomy [7]. In this case, the patient had undergone total hysterectomy. We accessed the transvaginal route from the beginning with a view of performing TVSE; thus, the intent of TVSE in the two reports differs.

The main advantages of this technique are as follows. It avoids the destruction of the abdominal wall and eliminates visible scars, decreasing the risk of abdominal hernia [11]. Furthermore, it also decreases postoperative pain and the risk of surgical site infection (SSI) [12]. In this case, the patient did not require any postoperative analgesics, and there was no SSI. Generally, obese patients tend to require a larger incision for specimen extraction, and obese patients are at an increased risk of developing SSI. In this aspect, TVSE is also useful for obese patients.

Complications of TVSE are reportedly infrequent [9, 10], but the risk of infection due to the transvaginal maneuvers is of concern. In our department, we irrigate the vagina and abdominal cavity with saline before and after extraction, and an Alexis® wound retractor and tissue collection bags are used for specimen extraction so as to protect the vagina and help prevent infections. We have not experienced any infections using TVSE in any of our other cases, including cases of colon, stomach, and liver cancer, as well as cancer of the small intestine. It is extremely important to ensure oncological suitability. No relapse specifically caused by TVSE has been reported for colon cancer [9, 12]; however, some reports have pointed out that to prevent peritoneal dissemination or delivery site metastasis, specimens should be put in a bag before extraction [6]. However, others claim that as long as oncological principles and procedures are followed, there should be no increase in the dissemination as a result of TVSE, and the validity of TVSE in early-stage uterine cervical cancer has been demonstrated in the field of gynecology [10]. In this case, the specimen was put in a collection bag for extraction. We also fragmented the spleen inside the bag for easier extraction. With this method, the extraction of larger solid organ specimens by TVSE is at least theoretically possible as long as there are no pathological problems.

In our department, we have performed NOSE mainly for colon cancer surgery. Some studies show that NOSE for colorectal cancer is feasible, safe, and oncologically acceptable for selected cases [13, 14]. TVSE enables us to extract relatively large specimens, the vagina being very elastic. TVSE is potentially adaptable to all types of abdominal carcinomas. TVSE being a technique under development, we generally perform TVSE only for carcinomas diagnosed in the absence of lymph node metastasis and serosa infiltration.

## Conclusions

We describe a case of splenic metastasis of ovarian cancer for which a complete laparoscopic splenectomy was performed in the supine position, with transvaginal specimen extraction. We eliminated the disadvantage of the lateral position using the supine position. Additionally, TVSE reduced the damage to the body wall, allowing an extremely minimally invasive operation. TVSE may become an option for even less invasive laparoscopic surgeries in the future.

## Consent

Written informed consent was obtained from the patient for the publication of this case report and any accompanying images. A copy of the written consent is available for review by the Editor-in-Chief of this journal.
